# Role of 6-hour, 12-hour, and 24-hour lactate clearance in mortality of severe sepsis and septic shock patients

**DOI:** 10.1186/cc14005

**Published:** 2014-12-03

**Authors:** V Herwanto, KC Lie, S Suwarto, CM Rumende

**Affiliations:** 1 Department of Internal Medicine, University of Indonesia, Jakarta, Indonesia; 2 Division of Tropical Medicine and Infectious Diseases, Department of Internal Medicine, University of Indonesia, Jakarta, Indonesia; 3 Division of Respirology and Critical Care, Department of Internal Medicine, University of Indonesia, Jakarta, Indonesia

## Introduction

Lactate is one of biomarkers used for risk stratification, resuscitation target, and death prediction in sepsis [1,2]. Interpretation of lactate clearance was proven more superior than single measurement to evaluate resuscitation adequacy and to determine prognosis [3,4]. This study aimed to find out whether mean differences of 6-hour, 12-hour, and 24-hour lactate clearance were observed between nonsurvivors and survivors of acute phase mortality in severe sepsis and septic shock patients.

## Methods

The study design was prospective cohort. Subjects were collected by consecutive sampling from the emergency department, hospital ward, and ICU at Cipto Mangunkusumo Hospital, Jakarta. Lactate levels were measured at 6, 12, and 24 hours, and subjects were subsequently followed to evaluate 3-day mortality. To determine their association with mortality, we used mean difference analysis of those three lactate clearance periods between nonsurvivors and survivors. In addition, to determine the cutoff value, we used receiver operator curve analysis.

## Results

Eighty-one subjects were included in this study. Eighty of 81 were followed until 12 hours, and 72 out of 80 were followed until 24 hours. Twenty-five subjects (31%) did not survive within 3 days of hospitalization. Only 24-hour lactate clearance had significant median difference (-17.0% in nonsurvivor vs. 15.2% in survivor group; *P *= 0.034). The best cutoff value for 24-hour lactate clearance was -6.0% (AUC 0.744, sensitivity 62.5% and specificity 87.5%, positive predictive value 58.8% and negative predictive value 89.1%, relative risk 5.39). From multivariate analysis, 24-hour lactate clearance was proven to be an independent predictor of mortality.

## Conclusion

Median of 24-hour lactate clearance was significantly lower in nonsurvivors of severe sepsis and septic shock patients. Its cutoff value was -6.0%.
